# Central obesity – the most common nutritional disorder in non-frail patients with chronic kidney disease 

**DOI:** 10.5414/CNP104S18

**Published:** 2025-11-28

**Authors:** Aljoša Kuzmanovski, Bojan Knap, Mojca Poljanec, Tjaša Bonča, Gašper Poljšak, Jelka Lindič

**Affiliations:** 1 Dietetics and Nutrition Service,; 2 Department of Nephrology, Division of Internal Medicine, University Medical Center Ljubljana,; 3 Faculty of Medicine, and; 4 Biotechnical Faculty, University of Ljubljana, Slovenia

**Keywords:** central obesity, chronic kidney disease, malnutrition, dietary habits, physical activity

## Abstract

Introduction: Patients with chronic kidney disease (CKD) often experience various nutritional disorders, leading to increased frailty, morbidity, and mortality. The aim of our study was to explore the impact of dietary habits and physical activity on body composition in non-dialysis CKD patients. Materials and methods: This cross-sectional study included 199 non-frail CKD patients (stages 1 – 5) from the University Medical Center Ljubljana. Participants completed a food frequency questionnaire, and anthropometric measurements were collected. Body composition was assessed using bioelectrical impedance analysis (BIA), and muscle strength was evaluated with handgrip dynamometry. Results: Central obesity was present in 79.9% of participants, and obesity based on body mass index (BMI) was found in 26.6%. Elevated fat mass was observed in 58.3% of patients. Sarcopenia was identified in 1%, and sarcopenic obesity in 0.5%. Based on the Global Leadership Initiative on Malnutrition (GLIM) criteria, 11.6% of patients were malnourished. Dietary analysis revealed too frequent intake of refined carbohydrates, red meat, and sweets, while intake of fruits and vegetables was not often enough. Dietary habits were suboptimal, with no significant differences between individuals with normal waist circumference and BMI and those with central obesity and elevated BMI. However, a significant difference was observed in physical activity, with those having central obesity engaging in less physical activity. Conclusion: Central obesity was the most prevalent nutritional disorder in non-frail CKD patients that was markedly more prevalent than obesity defined by BMI. Waist circumference was a more sensitive marker of increased fat mass determined with BIA than BMI. Inadequate dietary habits and insufficient physical activity were major contributing factors to the observed nutritional disorders in our CKD patients and call for long-term lifestyle modifications.

## Introduction 

Chronic kidney disease (CKD) is an increasingly common condition and a significant global public health challenge, affecting more than 10% of the world’s population [1]. Patients with CKD often experience various nutritional issues, especially sarcopenia, which plays a major role in increased frailty, morbidity, and mortality [2]. However, the prevalence and types of nutritional disorders in non-frail CKD patients remains underexplored. 

Dietary habits and physical activity are key determinants in the development of nutritional disorders, particularly in the context of CKD [3, 4]. In high-income countries, excessive consumption of refined carbohydrates, fats and sugars, combined with inadequate intake of nutrient-dense foods such as legumes and leafy green vegetables, directly contributes to the development of poor nutritional status, particularly manifesting as obesity and central obesity [5]. Obesity, especially central obesity, characterized by abdominal fat, is a well-established risk factor for the development of CKD and not only accelerates the progression of CKD, but also significantly increases the risk of cardiovascular complications [6, 7]. Physical inactivity exacerbates adverse changes in body composition and muscle strength, particularly in CKD patients who are already at risk of muscle wasting [8]. However, the specific interplay between dietary patterns, body composition, and muscle strength in patients with CKD remains underexplored. 

Consequently, we conducted this study to assess dietary habits, physical activity, nutritional status, and muscle strength in non-frail outpatient patients with CKD stage 1 – 5, not yet treated with replacement therapy, in order to identify key nutritional and lifestyle factors associated with nutritional disorders in this group of patients. 

## Materials and methods 

This cross-sectional, non-interventional study included 199 non-frail CKD patients (stages 1 – 5) monitored at the nephrology outpatient clinic of the Department of Nephrology, University Medical Centre Ljubljana, Slovenia. Patients who were unable to perform the 30-second sit-to-stand test, were excluded [9]. Additional exclusion criteria included patients who required assistance with mobility or had conditions that could interfere with reliable anthropometric or functional assessments. 

Patients were enrolled during routine follow-up visits. Anthropometric measurements, including body weight, height, and waist circumference, were obtained by trained personnel using standardized protocols. Obesity was defined as a body mass index (BMI) ≥ 30 kg/m^2^. Central obesity was determined by waist circumference thresholds of > 80 cm in women and > 94 cm in men [10]. 

Body composition was measured using a 4-frequency bioelectrical impedance analysis (BIA), device (Bodystat Quadscan 4000, BODYSTAT LTD, Brighton East Sussex, UK). Elevated fat mass was defined as ≥ 25% of fat mass in men and ≥ 35% of fat mass in women [11]. 

Muscle strength was assessed using handgrip dynamometer (Jamar Smart Hand Dynamometer, ASP Global, Austell, GA, USA) in seated participants with their elbows at 90 degrees, testing the dominant hand. Two measurements were taken; the higher value was recorded. Reduced muscle strength was defined as handgrip strength values < 16 kg for women and < 27 kg for men [12]. 

Malnutrition was assessed using the Global Leadership Initiative on Malnutrition (GLIM) criteria [13]. The GLIM criteria include three phenotypic criteria (non-subjective weight loss, low BMI, and reduced muscle mass) and two etiologic criteria (reduced intake or digestive malabsorption; inflammation or disease burden). The phenotypic criteria included: a) an unintentional weight loss > 5% for 6 months or > 10% for more than 6 months; b) a low BMI (a BMI of < 20 kg/m^2^ for individuals under 70 years or < 22 kg/m^2^ for those 70 and older); c) reduced muscle mass, which was classified as a fat-free mass index (FFMI) of < 15 kg/m^2^ in women and < 17 kg/m^2^ in men. Malnutrition was defined as at least one of the phenotypic and one of the etiologic criteria. 

Muscle mass was assessed using BIA. Reduced muscle mass (myopenia) was classified as a FFMI of < 15 kg/m^2^ in women and < 17 kg/m^2^ in men [14]. Sarcopenia was defined as the presence of reduced muscle mass and reduced muscle strength. 

Patients completed a structured food frequency questionnaire that included 11 food groups with 67 items to evaluate dietary habits: dairy products (7 items), fruit (3 items), vegetables (4 items), meat (4 items), fish (4 items), eggs (1 item), fats and nuts (8 items), breads and cereals (12 items), fast food (6 items), sugar and sweets (7 items), drinks (11 items). The participants were asked how often they consumed food items over the previous month. The grading system for evaluating the responses was designed to quantify the frequency of food consumption. For each food item, participants were assigned points based on their selected frequency: 3 points for several times per day, 1 point for daily, 0.7 points for 4 – 6 times per week, 0.29 points for 1 – 3 times per week, 0.06 points for 1 – 3 times per month, 0 points for rare or never. This grading system allowed for a numerical representation of dietary habits, facilitating statistical analysis and comparison of food consumption patterns. The questionnaire included information about whether they achieved 150 minutes of aerobic activity per week and whether they participated in strength exercises at least twice a week. 

Data analysis was performed using R version 4.4.1. For comparing differences between groups we used the independent samples t-test, for categorical variables the χ^2^-test. A p-value < 0.05 was considered statistically significant. 

The study protocol was approved by Medical Ethics Committee of the Republic of Slovenia (No. 0120-304/2023). All participants provided written informed consent before enrollment. 

## Results 

A total of 199 patients with CKD were included in the study, 50.8% were men. The mean age of patients was 57.4 ± 17.8 years, 41% (N = 82) of patients were older than 65 years. The average estimated glomerular filtration rate (eGFR) and serum creatinine were 56.9 ± 24.9 mL/min/1.73m^2^ and 138.9 ± 107.2 µmol/L, respectively. Age and CKD stage of the participants are presented in Table 1. 

All anthropometric measurements and handgrip strength of patients are present in Table 2. Among all the patients, 16.1% (N = 32) exhibited normal BMI and waist circumference. Obesity, as defined by BMI, was observed in 26.6% of patients (N = 53). The prevalence across the different stages of CKD was as follows: 13.2% in stage 1, 34% in stage 2, 39.6% in stage 3, 11.3% in stage 4, and 1.9% in stage 5. Central obesity was found to be more prevalent, affecting 79.9% of participants (N = 159). The distribution across the stages of CKD was as follows: 14.3% in stage 1, 33.8% in stage 2, 34.4% in stage 3, and 14.3% in stage 4, and 3.2% in stage 5. Importantly, all patients classified as obese according to BMI criteria also presented with central obesity. 

BIA revealed elevated fat mass in 58.3% (N = 116) of patients. The prevalence across the stages of CKD was as follows: 8.6% in stage 1, 36.2% in stage 2, 37.1% in stage 3, 16.4% in stage 4, and 1.7% in stage 5. Among patients with elevated fat mass, 96.6% (N = 112) also had central obesity (p < 0.001) and 42.2% (N = 49) were obese based on BMI (p < 0.001). On the other hand, 70.4% (N = 112) of patients who had central obesity had elevated fat mass measured by BIA (p < 0.001) and 33.3% (N = 53) of them were obese based on BMI (p < 0.001). Patients who were obese according to BMI had elevated fat mass measured by BIA in 92.5% (N = 49) (p < 0.001) and all of them had central obesity (N = 53) (p < 0.001). 

Sarcopenia was diagnosed in 1.0% (N = 2) of patients, (CKD stages 3 and 4). One patient (0.5%) with CKD stage 4 exhibited sarcopenic obesity. 

Myopenia was identified in 10.6% (N = 21) of patients, with a trend towards a higher prevalence among women (15.3%) compared to men (5.9%) (p = 0.06). The majority were in CKD stage 3 (N = 10), 4 patients stage 4, 4 patients stage 2, 2 patients stage 1 and 1 patient stage 5. Additionally, simultaneous myopenia and elevated fat mass were observed in 6% (N = 12) of participants (4 patients CKD stage 4, 4 patients stage 3, 4 patients stage 2). 

According to the Global Leadership Initiative on Malnutrition (GLIM) criteria, 23 patients (11.6%) were diagnosed with malnutrition. The majority of patients had CKD stage 3 (10 patients), 4 patients CKD stage 4, 4 patients stage 2, 4 patients stage 1, and 1 patient stage 1. 

**Figure 1:**
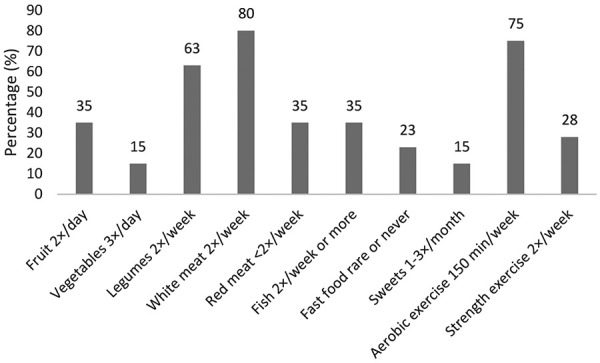
The percentage of patients with different food frequency intake, aerobic physical activity, and strength exercise according to general recommendations.

The percentage of patients with CKD with different food frequency intake, aerobic physical activity and strength exercise based on general recommendations is presented in Figure 1, and the frequency of consumption of individual foods is presented in Table 3. Vegetables, dairy products, fruits, and white flour products were most commonly consumed, on average at least daily or several times daily. 

Dietary analysis revealed no significant differences in dietary habits between obese patients (high BMI) with concomitant central obesity (26%, N = 52) and those with normal BMI and normal waist circumference (16%, N = 32). Both groups reported similarly and too frequent consumption of sweets, refined flour products, and red meat, coupled with insufficient frequency of fruit and vegetables intake. However, a significant difference was noted in physical activity levels: obese patients with central obesity were significantly less likely to engage in ≥ 150 minutes of weekly physical activity compared to patients with normal BMI and normal waist circumference (p = 0.003), while there was no difference in engaging in strength exercise, that was mostly insufficient. 

## Discussion 

The most common nutritional disorder in non-frail CKD patients in our study was central obesity, affecting 79.9% of our CKD patients and seemed to be somewhat more common in females than in males. Obesity, based on BMI, was much less common than the prevalence of central obesity and was observed in 26.6% of all participants, which is similar to the prevalence of obesity in the Turkish CKD population (29.2%) [15], yet higher than in the general population of Slovenia (19.9%) [16]. Elevated fat mass, measured with BIA, was observed in 58.3% of all patients. All patients with an increased waist circumference had elevated fat mass, as measured by BIA, making it a very sensitive marker of increased fat mass in our population. 

Waist circumference is known to be a more accurate predictor of elevated fat mass, particularly visceral fat, compared to BMI and allows for quick and easy detection of central obesity, which is linked to numerous adverse health effects and progression of CKD [17]. However, BIA remains crucial for assessing lean body mass and plays a key role in tracking changes in body composition during lifestyle interventions aimed at preventing muscle wasting. 

The identification of malnutrition in 11.6% of patients (mostly with CKD stage 3), based on the GLIM criteria, seems low compared to other studies. A systematic review by Rashid et al. (2021) [18] found that malnutrition prevalence in non-dialysis CKD patients ranged from 24 to 55.3%. We assume that this difference is due to the exclusion of frail patients in our study. Similarly, in our cohort, we encountered less patients with low muscle mass, as compared to other studies that reported a prevalence of 19.7% of patients, compared to our finding of 10.6% [19]. As observed in other studies, our research also found a higher prevalence of myopenia in women [20], bur preservation of muscle strength was comparable between male and female participants. Regardless of the differences with other studies, our results further emphasize the importance of using tools to assess nutritional risk in all stages of CKD. 

Reduced muscle mass coexisting with elevated fat mass, as seen in 6% of participants in our study, is clinically important, as it reflects the common occurrence of protein-energy wasting and changes in fat distribution in CKD patients. A combination of reduced muscle mass and increased body fat indicate sarcopenic obesity, a condition strongly linked to worse clinical outcomes [21]. Bellafronte et al. [22] (2021) found that the prevalence of sarcopenic obesity ranged from 1 to 16% in patients with CKD, depending on the diagnostic criteria, but again, our results (1 patient, 0.5%) cannot be directly compared due to the exclusion of frail patients. 

Dietary habits in this cohort revealed too frequent intake of refined carbohydrates, sweets, and red meat, while the consumption of nutrient-dense foods such as fruits and vegetables was not often enough. It has been well established that dietary patterns high in processed and animal-based foods, and low in plant-based foods, are associated with obesity, an increased risk of CKD progression, and cardiovascular disease. This is concerning, as it indicates that many CKD patients may not be adhering to healthy lifestyle and dietary recommendations that promote kidney health [5]. 

Physical activity, including strength exercises, has been shown to help prevent muscle wasting, reduce waist circumference, and improve body composition in CKD patients [4]. Our findings indicate that physical activity is an important factor in maintaining normal weight and body composition even when dietary habits are not as healthy as recommended. However, 75% of patients met the 150 minutes of weekly aerobic exercise, and only 28% regularly participated in strength exercises. Resistance training is particularly important for CKD patients, as it can help address inflammation, metabolic disturbances, and protein-energy wasting, especially in the later stages of CKD. Simple home-based exercise programs have been shown to be effective in reducing muscle atrophy in pre-dialysis CKD patients, preserving muscle mass and strength, and preventing weight loss due to malnutrition [23]. 

We conclude that non-frail CKD patients seem to experience a range of nutritional disorders, with central obesity being the most prevalent and easily identified with waist circumference measurements. Recognizing nutritional disorders is crucial for all CKD patients, including those in the early stages, who stand to benefit the most from lifestyle interventions. Key contributors to nutritional disorders in our CKD population include inadequate dietary habits and insufficient physical activity. Our findings emphasize the importance of long-term lifestyle modifications and a multidisciplinary approach, involving nephrologists, dietitians, kinesiologists, and psychologists, to optimize patient outcomes. 

## Acknowledgment 

For the study investigations, we thank the diligent nurses of the nephrology outpatient clinic. 

## Authors’ contributions 

Conceptualization, A.K., J.L., and B.K.; methodology, A.K., J.L., and B.K.; acquisition of patient data, M.P., G.P., and T.B.; validation, A.K.; formal analysis, J.L.; writing – original draft preparation, AM.; writing – review and editing, A.K., J.L. All authors read and approved the final version of the manuscript. 

## Funding 

The project was funded through research funds of the University Medical Center Ljubljana, No. 20240079. 

## Conflict of interest 

The authors declare no conflict of interest for the study. 

**Table 1.** Demographic and clinical characteristics of the participants.

Parameter	Men (N = 101)	Women (N = 98)	Total (N = 199)
Age			
18 – 30	11 (10.9%)	9 (9.2%)	20 (10.1%)
31 – 50	25 (24.8%)	24 (24.5%)	49 (24.6%)
51 – 65	22 (21.8%)	26 (26.5%)	48 (24.1%)
65 and more	43 (42.5%)	39 (39.8%)	82 (41.2%)
CKD stage			
1	13 (13.5%)	21 (21.6%)	34 (17.6%)
2	23 (24.0%)	35 (36.2%)	58 (30.1%)
3	35 (36.5%)	30 (30.9%)	65 (33.6%)
4	19 (19.7%)	8 (8.2%)	27 (14%)
5	6 (6.3%)	3 (3.1%)	9 (4.7%)

**Table 2.** Anthropometric measurements and handgrip strength of patients.

Parameter	Men (N = 101)	Women (N = 98)	Total (N = 199)	p-value
Height (cm)				
Mean	175.64	164.14	169.98	< 0.005
SD	± 10.76	± 6.63	± 10.64	
Weight (kg)				
Mean	86.42	73.24	79.93	< 0.005
SD	± 16.99	± 13.51	± 16.70	
Waist circumference (cm)				
Mean	103.23	96.03	99.65	< 0.005
SD	± 12.45	± 13.88	± 13.63	
Waist category				
Normal waist circumference	26 (25.7%)	14 (14.3%)	40 (20.1%)	0.0502
High waist circumference	75 (74.3%)	84 (85.7%)	159 (79.9%)	
BMI (kg/m^2^)				
Mean	28.15	27.25	27.71	0.554
SD	± 6.29	± 5.21	± 5.79	
BMI category				
< 30	75 (74.3%)	71 (72.4%)	146 (73.4%)	0.905
≥ 30	26 (25.7%)	27 (27.6%)	53 (26.6%)	
Handgrip strength (kg)				
Mean	45.5	30.69	41.17	< 0.005
SD	± 11.52	± 5.9	± 12.83	
Handgrip category				
Normal strength	96 (96%)	98 (100%)	194 (98%)	0.137
Low strength	4 (4%)	0 (0%)	4 (2%)	

**Table 3.** The average frequency of consumptions of individual foods according to grading system of scoring per category: several times per day – 3 points, daily – 1 point, 4 – 6 times per week – 0.7 points, 1 – 3 times per week – 0.29 points, 1 – 3 times per month – 0.06 points, rare or never – 0 points.

Food group	Average frequency	Average score (SD)
Dairy products	Daily	1.71 (165)
Vegetables	Daily	1.94 (130)
Leafy green vegetables	4 – 6×/week	0.81 (0.66)
Cruciferous vegetables	1 – 3×/week	0.31 (0.32)
Legumes	1 – 3×/week	0.33 (0.49)
White meat	1 – 3×/week	0.35 (0.30)
Red meat	1 – 3×/week	0.26 (0.23)
Processed meat	1 – 3×/week	0.14 (0.20)
Fruits	Daily	1.65 (1.23)
Fish	1 – 3×/week	0.31 (0.47)
Eggs	1 – 3×/week	0.34 (0.42)
Olive oil	4 – 6×/week	0.78 (0.74)
Butter	1 – 3×/week	0.42 (0.60)
Unsalted nuts	1 – 3×/week	0.16 (0.41)
White flour products	Daily	1.04 (0.98)
Whole grain products	4 – 6×/week	0.97 (0.85)
Fast food	1 – 3×/week	0.20 (0.32)
Sugar	4 – 6×/week	0.99 (1.17)
Water	Daily	2.57 (0.83)
